# The temporal transition zone: A gradual approach to a subjective set‐point within the three‐second time window

**DOI:** 10.1002/pchj.755

**Published:** 2024-04-28

**Authors:** Chen Zhao, Nan Mu, Jiyuan Zhang, Yan Bao

**Affiliations:** ^1^ Institute of Medical Psychology Ludwig Maximilian University Munich Germany; ^2^ School of Psychological and Cognitive Sciences Peking University Beijing China; ^3^ Beijing Key Laboratory of Behavior and Mental Health Peking University Beijing China

**Keywords:** duration reproduction, temporal perception, time window

## Abstract

Even though in physics “time” is considered to be continuous, how the brain and mind deal with time might be different. It has been proposed that in cognition, time windows provide logistic platforms for information processing, such as the low‐frequency 3‐s time window. The following series of behavioral experiments may shed light on the dynamics within such a time window. Using a duration reproduction paradigm, we first replicated a pattern of reproduced duration observed in a previous single‐case study. Specifically, the reproduction increases as the pause between standard duration and reproduction increases, but only within the time window of some 3 s; when the pause goes beyond 4 s, the reproduction reaches a plateau of a subjective set‐point. This increasing phase is named the “temporal transition zone.” Three more experiments were performed to test the features of the transition zone as a low‐frequency time window. It is also observed with different standard durations (2, 3, 4.5 s, in Experiment 2), and even when the frequency of the auditory stimuli was different in standard and reproduction (300 Hz in standard duration and 400 Hz in reproduction, in Experiment 4). The transition zone was observed only with pause durations of 2 to 3 s; when the shortest pause duration was 5 s, the transition zone was no longer observed, and the reproduction was stable at the subjective set‐point (in Experiment 3). Taken together, we suggest that the temporal transition zone indicates a pre‐semantic logistic platform to organize and process the information flow; in such a time window of some 3 seconds, the identity of an ongoing event is substantiated, building the “subjective present.”

## INTRODUCTION

**FIGURE 1:**
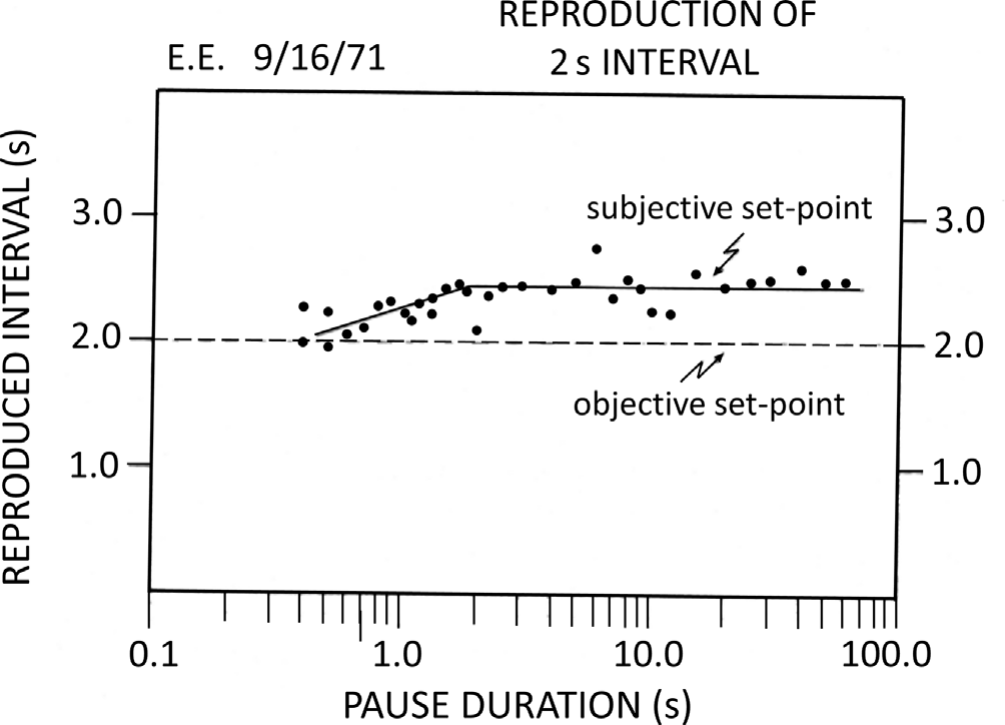
Reproduced duration of a single subject with pause durations logarithmically manipulated from 0.5 to 100 s (Pöppel, 1973).

Time, in which information flow is embedded, has been intensively studied in the cognitive sciences. In classical physics (Newton, 1969) and implicitly at the psychological level, it is assumed that time is continuous; in the mainstream of cognitive sciences, time is studied with a similar approach to that used with sensory inputs such as visual or auditory stimuli, even though we possess no sensors for time as such (Ulbrich et al., 2007). With this perspective, time has been treated as a cognitive content, namely how we perceive durations. Various paradigms have been developed, such as duration comparison, production, estimation, and reproduction (Mu et al., 2023; Pöppel, 1972; Szelag et al., 2002), and many previous studies have employed these paradigms with various modifications. One study that varied the temporal structure in the reproduction paradigm revealed an intriguing finding (Pöppel, 1973). In a single‐case study, a subject was asked to reproduce a duration of 2 s, not immediately after perceiving it, but after several seconds. The waiting time, later referred to as the pause duration, varied from 1 to 100 s. The reproduction from this participant is illustrated in Figure 1. At first, what attracted the most attention was the long plateau after a certain length of pause duration, which is considered to be a stable subjective perception of a duration. Such a subjective set‐point is always longer than the actual duration when the latter is shorter than about 3 s (Mu et al., 2023).

Later, in reviews of the early work, it was noticed that there is an increasing phase before the reproduction reaches the plateau, which suggests that there might be a positive correlation between the reproduction and pause duration within a few seconds. This phenomenon interests us very much as it echoes a theory of temporal organization in cognition (e.g., Zhao et al., 2022). Without denying that time can be perceived as a cognitive content such as duration, we take time as a logistic frame to organize information. The temporal logistic is possibly realized by time windows, including high‐frequency (30–40 ms) and low‐frequency (2–3 s) ones, according to the hierarchical model of time perception (Pöppel, 1997). The low‐frequency 3‐s time window, in particular, has been endorsed by both behavioral and neuro‐recording studies in many fields, including duration perception, in‐attentional information processing, aesthetic judgment, and linguistic performance (Bao et al., 2015; Wang et al., 2015; Zhao et al., 2018; Yu & Bao, 2020); aside from studies with healthy participants, studies with autistic children also endorse the low‐frequency time window (Szelag et al., 2004). By providing a platform to build a subjective present, the 2–3‐s time window is considered to be a pre‐semantic logistic mechanism, independent of cognitive content. Only when the proper logistic functions, such as temporal structural organization, are complementarily working with the content functions can normal cognition be preserved (Bao et al., 2022; Zhao et al., 2022).

The single‐case study provides a practical and critical paradigm first to verify the low‐frequency time window and then to further investigate the mechanism by manipulating various parameters. Therefore, we adopt and manipulate the pause‐duration paradigm to systematically examine the pattern of increasing reproduction with a longer pause duration. We believe, on the behavioral level, that the experiments could provide more evidence supporting the low‐frequency time window and lead us to thinking about the underlying mechanism, not only psychological ones but also neural ones to help us develop a mature paradigm to explore neural markers.

## EXPERIMENT 1

First, we designed a simple experiment applying the duration reproduction paradigm to examine whether we could replicate the pattern of reproduction as observed with a single subject previously (Pöppel, 1973).

### Method

#### Participants

Twelve Chinese university students aged between 18 and 25 years old participated in the experiment (2 males and 10 females). All participants were right‐handed and had normal hearing without any history of mental or neurological disease.

#### Design and apparatus

**FIGURE 2:**
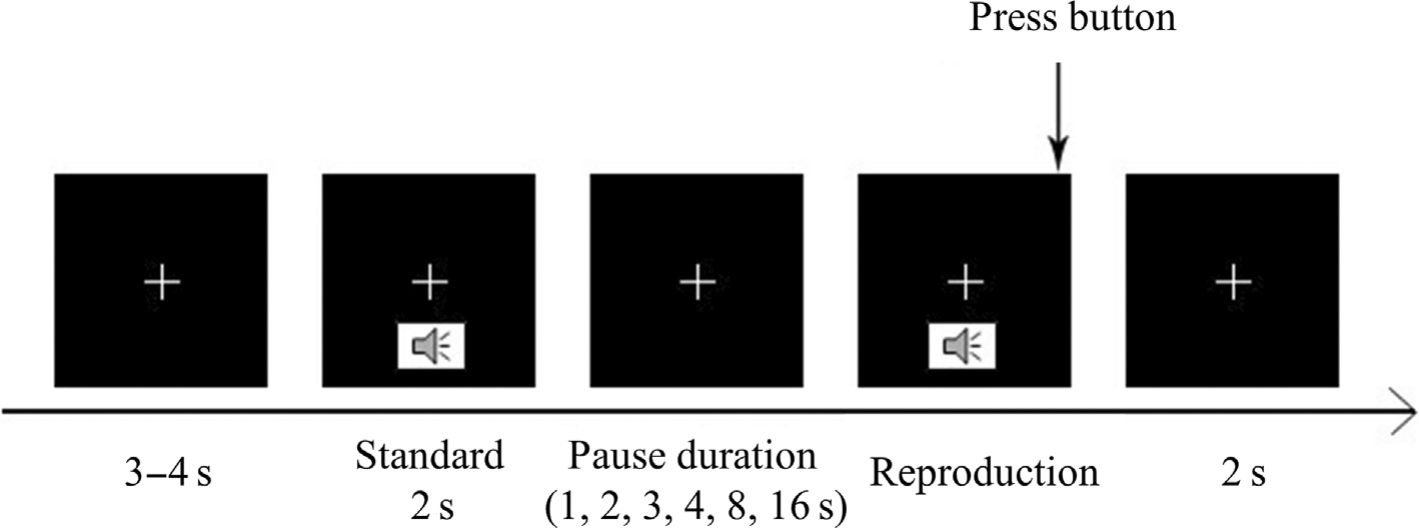
The paradigm of duration reproduction in Experiment 1 (for details, see text).

We applied the paradigm of duration reproduction. The task started with a fixation cross (0.8°), which was presented for 3–4 s. Then, the standard duration of 2 s was delivered by a pure tone at 300 Hz with binaural presentation. Before the reproduction, there was a pause duration, which was manipulated at five levels (1, 2, 4, 8, or 16 s). Then, the reproduction started with the pure tone, and participants needed to terminate the tone with the space bar to indicate their reproduction (see Figure 2). Thus, the only independent variable was the pause duration, which had five levels. Each level was repeated for 20 trials, and the resulting 100 trials were randomly distributed into four sessions. The experiment was programmed with Matlab 2019a and Psychtoolbox (Kleiner et al., 2007) and was performed on a Dell T3610 computer. The sound adapter Blaster Audigy 5 and an auditory stream input/output interface were applied to improve the timing accuracy of the auditory stimuli. Before the experiment, all participants adjusted the sound volume to a comfortable level and got used to an in‐ear headphone.

#### Procedure

All participants first signed the consent form before starting the experiment. During the whole experiment, participants sat in front of the screen with their heads on a chinstrap. At the beginning, they were asked to complete a practice session containing 10 trials to get familiar with the task. The 10 practice trials included all five conditions, each repeated twice. The following formal experiment had four sessions interleaved by 3‐min break intervals. Participants were informed several times that they should only feel and remember the duration, and any kind of counting was forbidden. This has been shown to be the best and easiest way to avoid the counting strategy in previous research (Rattat & Droit‐Volet, 2012).

### Results

**FIGURE 3:**
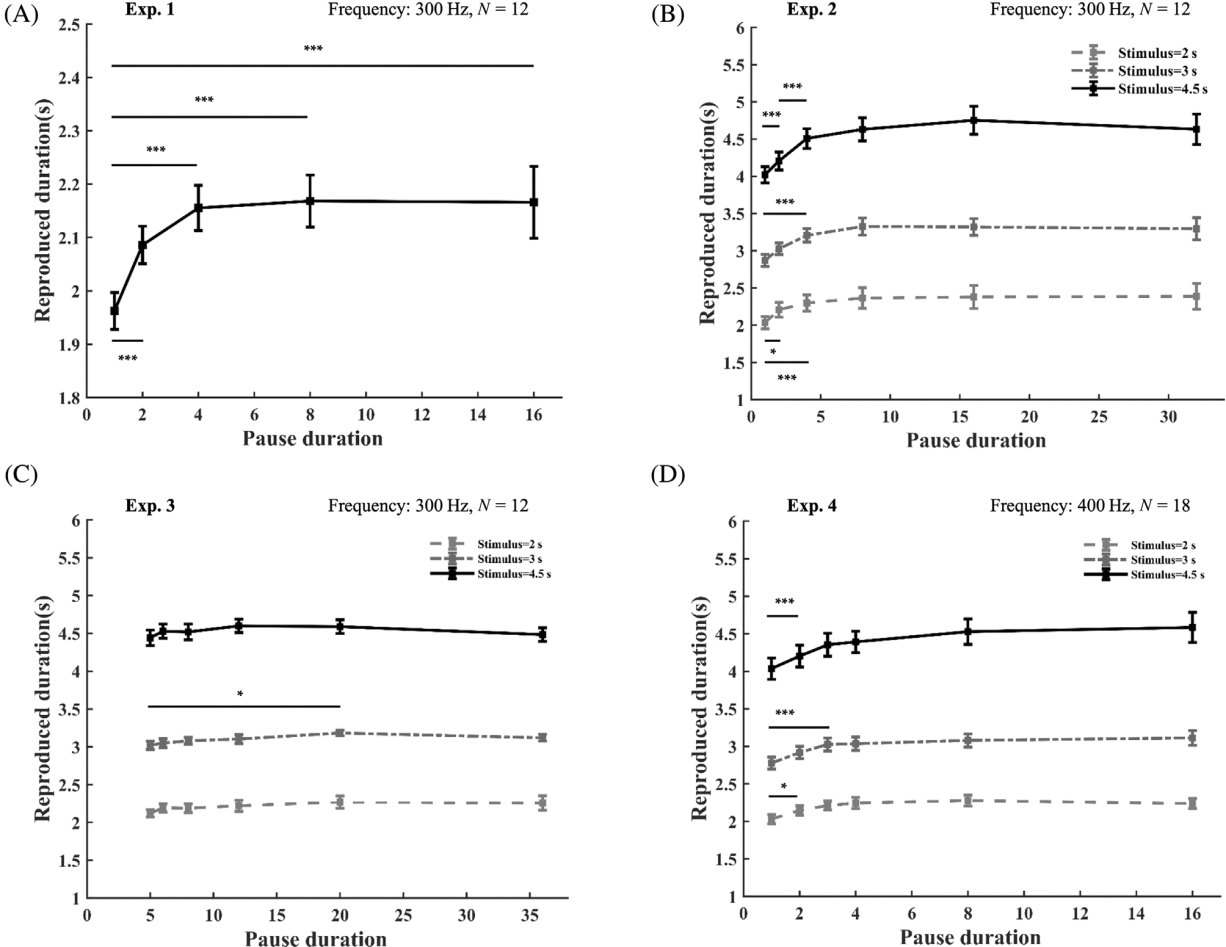
Reproduced durations after different pause durations in four experiments (for details, see text). The frequency indicated is the frequency of the auditory stimuli in the reproduction phase.

For data screening, we created both a scatter graph and a box plot for each participant. Responses over a double standard deviation were recognized as outlier trials and were excluded from the dataset. For statistical analysis, a one‐way repeated measure analysis of variance (ANOVA) was performed in SPSS 22.0. The results revealed a significant effect of pause duration (*p < *.001, *F*(4,68) = 10.44). Post hoc paired comparison showed that the reproduction was shorter when the pause duration was 1 s (mean [*SD*] = 1.94 [0.33]) compared with other levels (*p < *.001); and the reproduction was also shorter when the pause duration was 2 s (mean [*SD*] = 2.09 [0.27]) compared with longer levels (*p < *.001), as illustrated and marked in panel A of Figure 3.

### Discussion

The results of the first study replicated the behavioral pattern from a previous study (Pöppel, 1973). The behavioral results show a similar pattern of reproduction: an increasing phase followed by a plateau. Specifically, when the pause duration is within 4 s, the reproduction increases as the pause duration increases, while with longer pauses, the reproduction tends towards a stable value, which is slightly longer than the standard duration.

It is intriguing to observe that when the pause is less than 3 s, the reproduction is consistently lower than the plateau, and the plateau is considered as a stable subjective set‐point. Not only this, but also based on the results, it seems that in the increasing phase, a longer pause duration is reliably associated with a longer reproduction. Within such a time zone, even though the standard stimulus is perceived, which should be endorsed by the stable subjective set‐point, it cannot be reproduced as such. Based on such characteristics, we refer to the time zone of increasing reproductions as *the transition zone*. We believe that it represents a pre‐semantic logistic frame of information processing and we would like to further examine the generality of this phenomenon.

Thus, in the following experiments, we tested several factors independently, namely the standard duration (Experiment 2), pause duration (Experiment 3), and frequency of the auditory stimulus (Experiment 4). We are interested in the robustness of the transition‐zone effect of duration reproduction when the parameters in the experimental paradigms are being manipulated. We believe that this series of experiments provides the litmus test to demonstrate that the transition zone represents a pre‐semantic temporal logistic function for cognitive contents.

## EXPERIMENT 2

As the 3‐s time window has been demonstrated to be embedded in a cluster of cognitive phenomena, we wondered whether the transition‐zone effect would be specific to a duration within 3 s. The results from Experiment 1 are limited by the standard duration of 2 s to be reproduced. Thus, in the second experiment, we expanded the range of the standard duration to examine the transition‐zone effect for other durations beyond 2 s. The tested standard duration has three levels, namely 2, 3, and 4.5 s. Such levels were chosen to cross the critical threshold of 3 s, as previous studies and theories suggest 3 s to be a differential factor in cognitive phenomena. Furthermore, the difference ratio between levels is equalized, as 3/2 is equal to 4.5/3.

### Method

#### Participants

Twelve students from Peking University volunteered to participate in this experiment, 10 of whom were female. Their ages were between 18 and 25 years old, and all of them had normal hearing and were right‐handed.

#### Design and apparatus

Based on the design of Experiment 1, Experiment 2 employed three standard stimuli to be reproduced (2, 3, and 4.5 s). Participants were asked to come for the experiment on three separate days, and only one standard duration was tested each day. The sequence of the tests with the three standard durations was counter‐balanced among all participants. Pause durations varied from 1 to 32 s (1, 2, 4, 8, 16, 32 s), with one more pause duration (32 s) than in Experiment 1. Each pause duration was repeated 18 times for each standard duration. The total of 108 trials were randomly distributed into six blocks, with breaks interleaved. The equipment used in Experiment 2 was identical to that in Experiment 1.

#### Procedure

All participants first consented to participate and then were asked to come in on three different days for the three standard duration conditions separately. The procedure for each condition was the same as in Experiment 1. A practice session consisting of two trials with each pause duration was always delivered before the formal test.

### Results

Again, for data screening we applied both a scatter graph and a box plot for each participant. Responses over double the standard deviation were recognized as outlier trials and were excluded from the dataset. The remaining data were subjected to a repeated two‐way ANOVA in SPSS 22.0.

As visualized in panel B of Figure 3, there is a significant interaction between the standard duration and the pause duration (*F*(10,130) = 4.54, *p < *.01). The main effects of the standard duration (*F*(2,26) = 280.38, *p < *.001) and the pause duration (*F*(5,65) = 15.12, *p < *.001) are both significant. Post hoc analysis was performed to further clarify the interaction. For each level of standard duration, we examined the simple effect of the pause duration, specifically in the window of the transition zone. When the standard duration was 2 s, the reproduction with the 1‐s pause duration (mean [*SD*] = 2.03 [0.39]) was shorter than that of the 2‐s pause duration (mean [*SD*] = 2.20 [0.44], *p < *.05) and the 4‐s pause duration (mean [*SD*] = 2.30 [0.46], *p < *.001); the reproduction of the 2‐s pause duration was marginally shorter than that of the 4‐s pause duration (*p* = 0.073). For the standard duration of 3 s, the reproduction with the 1‐s pause duration (mean [*SD*] = 2.87 [0.46]) was shorter than that with the 4‐s pause duration (mean [*SD*] = 3.21 [0.49], *p < *.001). For the standard duration of 4.5 s, the reproduction with the 1‐s pause duration (mean [*SD*] = 4.01 [0.56]) was shorter than that with the 2‐s pause duration (mean [*SD*] = 4.21 [0.62], *p < *.001); and the reproduction with the 2‐s pause duration was shorter than that with the 4‐s pause duration (mean [*SD*] = 4.49 [0.68], *p < *.001).

### Discussion

In Experiment 2 we manipulated the standard duration, extending it over the 3‐s time window up to 4.5 s. The results revealed the same pattern of reproduction across different pause durations. Specifically, independent of the length of the standard duration, the reproduction increases as the pause duration increases within the transition zone of 3–4 s; when the pause duration exceeds the transition zone, the reproduction is stable around the subjective set‐point.

Before being confirmative about the robustness of the transition‐zone effect, alternative possibilities need to be clarified. In the following Experiment 3, we applied the same paradigm while changing the pause durations to a different set. One of the confounding possibilities is that the transition zone does not reflect a critical time window of some 3 s, but is an artifact of the paradigm design reflecting an initial effect of the time window. Specifically, it could be argued that within any shortest pause duration in an experiment, the transition zone might be observed, irrespective of the time window of 3 s. Thus, we systematically prolonged the pause durations, starting from a longer pause time of 5 s, to check whether such a design would induce the transition‐zone effect as well.

## EXPERIMENT 3

### Method

#### Participants

Twelve university students aged between 19 and 25 years old were recruited, five of whom were female. All participants had normal hearing and were right‐handed.

#### Design

The experimental design was the almost the same as in Experiment 2. We also manipulated the standard duration (2 s vs. 3 s vs. 4.5 s) and pause duration, but here the pause durations were changed to 5, 6, 8, 16, 20, and 36 s to go beyond the 3‐s time window. The task had the same duration reproduction paradigm.

#### Procedure

The experimental procedure was as the same as in Experiment 2. After consenting to participate, all participants were asked to come in on three different days to do the reproduction task with the three standard durations separately.

### Results

Data screening was performed before analysis. Abnormal responses and outliers were excluded. Then the data were subjected to a repeated‐measured ANOVA in SPSS 22.0.

As illustrated in panel C in Figure 3, there is no interaction between the variable of standard duration and pause duration, while the main effects are significant. For the standard duration effect, *F*(2,22) = 382.33, *p < *.001; for the pause duration effect, *F*(5,55) = 6.97, *p < *.001. A post hoc analysis of the pause duration on different levels of standard duration showed that only on the level of 3‐s standard duration, the reproduction with 5‐s pause duration was longer than that with 20‐s pause duration, *p < *.05.

### Discussion

When we systematically increased the pause durations in the experiment, specifically using 5 s as the shortest one, the transition‐zone effect disappeared. As the results revealed, the reproduction of all levels of the standard duration had reached its stable subjective set‐point of equivalence and did not increase as the pause duration was extended. That is to say, the transition‐zone effect disappeared in such an experimental design, meaning that it was not induced by the ordinal design of pause durations, but rather is embedded in the time window of some 3 s. A further necessary question to be tested was whether the transition‐zone effect would persist when changing the physical features of the standard duration. We found this indeed to be the case by changing the length of the standard duration in Experiment 2. In addition to that, we also need to test whether the transition‐zone effect is independent of changes to the acoustic features of the sound stimuli.

## EXPERIMENT 4

### Method

#### Participants

Eighteen university students volunteered to participate; their age ranged between 18 and 25 years, and 12 of them were female. They all had normal hearing and were right‐handed.

#### Design

The design of Experiment 4 was the same as that of Experiment 2. The standard duration had three levels, namely 2, 3, and 4.5 s, and the pause durations were 1, 2, 3, 4, 8, and 16 s. However, in Experiment 4, the frequency of the auditory stimuli in the reproduction phase was changed to 400 Hz, compared with the stimulus presentation phase of 300 Hz.

#### Procedure

All participants first consented to participate, and then were asked to come in on three different days for the three standard durations respectively. The practice trials and the formal experiment were the same as for the previous experiments.

### Results

Again, the data were subjected to a repeated‐measure two‐way ANOVA analysis. The results are presented in panel D of Figure 3. As can be seen, the interaction between the standard duration and the pause duration was significant, *F*(10,170) = 5.75, *p < *.001. Respectively, the main effect of the standard duration (*F*(2,34) = 272.84, *p < *.001) and the pause duration (*F*(5,85) = 39.57, *p < *.001) were also significant.

Further simple‐effect tests were done to examine the transition zone effect. When the standard duration was 2 s, the reproduction with 1‐s pause duration (mean [*SD*] = 2.03 [0.39]) was significantly shorter than that with 2‐s (mean [*SD*] = 2.20 [0.44], *p < *.05) and longer pause durations. When the standard duration was 3 s, the reproduction with 1‐s pause duration (mean [*SD*] = 2.87 [0.46]) was shorter than that with 3‐s (mean [*SD*] = 3.03 [0.33], *p < *.001) and longer pause durations. And when the standard duration was 4.5 s, the reproduction with 1‐s pause duration (mean [SD] = 4.05 [0.52]) was shorter than that with 2‐s (mean [SD] = 4.21 [0.54], *p < *.001) and longer pause durations.

### Discussion

Changing the frequency of the auditory stimuli does not affect the essence of the transition zone, as it can be seen that there is still an increasing trend of the reproduction as the pause duration gets longer in the time window of some 3 s. The results support the hypothesis that the transition‐zone effect is independent of the physical feature of the content – in this case the duration to be perceived. One could still argue that other auditory properties might have an influence, such as loudness and pitch of the stimuli. However, taking the results from Experiment 2 and Experiment 4 together, we are convinced that the transition‐zone effect is not determined by any specific cognitive content delivered. Rather, we believe that the transition‐zone effect is a temporal logistic function. It could, however, be interesting in future research to test whether monaural delivery would make a difference, because a previous study has suggested that the two hemispheres of the brain exhibit different potentials in processing auditory information (Zhao & Bao, 2023).

## GENERAL DISCUSSION

Taking the four experiments together, we observed under different conditions a consistent pattern of reproduction, including an increasing phase, *the transition zone*, and a plateau of subjective set‐point. Importantly, our independent manipulation of the length of standard duration, the setting of pause durations, and the frequency of the auditory stimuli during reproduction convinced us that the transition‐zone effect revealed a time window of some 3 s as a logistic platform to dynamically establish the identity of a target (Pöppel & Bao, 2014).

In Experiment 2, with three different levels of standard duration, the transition‐zone effect persists and is compatible with the low‐frequency time window from the hierarchical model. The results demonstrate that there is a fundamental difference between content‐wise time, as duration, and logistic time, an operating framework of information. The transition zone is considered to represent a logistic frame for content to be processed and established into a stable subjective identity (Zhao et al., 2022). In the case of the duration–reproduction paradigm, the content happens to be different durations. The two distinct properties of time, content versus logistic, are very likely to correspond to the nature of the hierarchical model of time perception, which proposes that the time windows represent the logistic operation of cognitive functions. Specifically, Experiment 3 proves that such a time window is exclusively in the range 2–3 s. In future research, we would like to examine whether the reproduction of other sensory content, such as brightness and loudness, also shows a transition zone. Further, in Experiment 4 we changed only the auditory frequency in the reproduction phase. Even though participants heard a different pitch for the standard duration and the reproduction, the reproduced duration still varied with the pause duration. Such an observation demonstrates that the transition zone is pre‐semantic, which means that temporal organization is not determined by cognitive content; rather it determines the temporal structure of such cognitive processes (Bao et al., 2022).

It needs to be noted that besides the four experiments employing the auditory paradigm, the transition‐zone effect should also be tested in a behavioral study using a duration–reproduction task in another modality, such as with visual presentation. We hypothesize that the transition‐zone effect is a robust behavioral observation on human cognitive performance, as we propose it to be a pre‐semantic logistic function. Then we are faced with the following reasonable and important question of “why”: What is the neural mechanism underlying this phenomenon, and what could be the psychological and ecological meaning?

Based on the behavioral results, it would be radical to speculate about any processes on the neural level. As efforts are necessary to identify neural markers for the transition zone, here we could provide a psychological explanation for the existence of the transition zone. As elaborated above, we believe that the overlapping between the transition zone and the low‐frequency time window of some 3 s is not a coincidence. Time is a fundamental dimension of the physical world, and the brain and the mind need to organize all kinds of information to make sense. It has been proposed that identity is a way out of the jungle of time (Pöppel, 2009; Zhou et al., 2014). In the low‐frequency time window, the mind is building a holistic subjective present: perceived sensory inputs, emotional reactions, and elicited memories are capsuled in a temporal unit and then connected to give the illusion of temporal continuity (Zhao et al., 2022). The transition‐zone effect reveals the dynamic process of the building up of the identity of a perceived object. The reproduction increases towards the plateau in the transition zone, even though at some point in between it is the same as the physical duration, it will progress towards its psychological identity. One curious characteristic of such a logistic phenomenon is that the subjective identity seems to be “pre‐determined,” as the subjective plateau is set as the goal at the onset of the perception. Certainly, we are aware that the limited number of behavioral experiments prevents us from making extensive speculations, but the consistency of the pattern in the temporal organization of cognitive functions, which has been documented in various subfields, requires further investigation and thinking.

## CONFLICT OF INTEREST STATEMENT

The authors declare that there are no conflicts of interests.

## ETHICS STATEMENT

The study was approved by the Ethical Committee of the School of Psychological and Cognitive Sciences, Peking University.
